# Evaluating a computerized CCS model against current technologies to optimize environmental conservation for the Patuakhali power plant in Bangladesh

**DOI:** 10.1016/j.heliyon.2024.e37107

**Published:** 2024-08-28

**Authors:** Md. Nasirul Islam, Md. Shameem Hossain, Mohammad Mujtaba Hasan, Shehoba Yasmin, Prodeepta Neogi

**Affiliations:** a Department of Petroleum and Mining Engineering, Military Institute of Science and Technology (MIST), Dhaka-1216, Bangladesh; b Department of Energy Science and Engineering, Khulna University of Engineering & Technology (KUET), Khulna -9203, Bangladesh

**Keywords:** Patuakhali power station, Carbon emission, Greenhouse gas, Sequestration, Reduction, Carbon capture, Emissions

## Abstract

Bangladesh's burgeoning focus on power generation has prompted the government to implement ambitious plans to install power plants. Among these developments is the impending operation of a 2∗660 MW coal-power station in Patuakhali, which will operate at the end of the month in December 2024. The proposed technology addresses concerns about CO_2_ emissions from a plant, potentially causing health issues and threatening plant biodiversity, but may present challenges compared to other technologies. Monoethanolamine (MEA), eutectic, and potassium taurate are potential solvents for CO_2_ capture in coal power plants due to their power absorption rate, capacity, and resilience to oxidative as well as thermal degradation. However, the significant challenges include corrosiveness, solvent loss, and high energy demand. By contrast, advanced research includes fixed and capture level reduction operating modes for carbon dioxide removal in natural gas combined cycle power plants, which is appropriate for use in natural gas combined cycle (NGCC) power plants where further research is needed for coal-fired power plants. The current generation of CO_2_ removal equipment, such as electrostatic precipitators (ESP) and flue gas desulphurization units (FGD), can remove CO_2_ at 99 % and 80 %–99 %, respectively. These devices have several serious drawbacks, including high water consumption, high costs, complex waste management, and operational errors. Additionally, equipment must be modified to increase efficiency and maximize heat rate. Notably, the moisture content in coal must be reduced from 0.6 to 5.9 %, heat must be recycled from 1.2 to 3.6 %, the steam turbine loop must be improved from 2 to 4.5 %, and advanced controls and sensors must be replaced or used up to 1.5 times.

Our study, utilizing an established operational model sanctioned within the country and assessment, revealed an approximate daily carbon emission of 4.806 million kilograms from the power plant. Employing the Sundarbans' sequestration rate, we calculated a carbon tolerance level of around 4.2 million kilograms daily for the plant area. This study also highlights the potential of computerized carbon capture and storage (CCCS) technology to significantly reduce emissions in the Sundarbans, which have nearly zero levels. It compares a computerized CCS model with an existing model, estimating over 90 % reduction considering 10 % mechanical faults. Implementing a computerized system can reduce CO_2_ leaks, risks, operational efficiency, costs, and policy compliance. It ensures the security of carbon capture, transportation, and storage processes, balancing environmental preservation and economic development. Advanced technologies can reduce emissions to zero, and the captured carbon can be used for petroleum-enhanced oil recovery techniques, which are briefly described. It also offers economic benefits and carbon credits, improving air quality and ocean health by mitigating pollutants and CO_2_ emissions.

## 1 Introduction

A thermal power plant that uses coal combustion to produce electricity is known as a coal-fired power station or coal power plant. More than 2400 coal-fired power plants with a combined capacity of more than 2000 GW exist worldwide [1]. They produce roughly one-third of the world's electricity, but they also lead to the majority of premature deaths and numerous illnesses due to air pollution. Bangladesh released a document titled revisiting power system master plan (PSMP) 2016 in November 2018 [2]. It featured two phases for the proposed Patuakhali power plant. The first deployment phase is expected by December 2024, with a targeted capacity of 1320 MW. By December 2030, the second phase was expected to have 1320 MW and begin operations [3]. The plant was designed to run at ultra-supercritical levels using imported coal from Australia, India, or Indonesia, amounting to about 12,000 tonnes of coal per day [4]. A transmission line would be constructed by the government-owned West Zone Power Distribution Company Limited of Bangladesh in order to connect the proposed plant to the Patuakhali sub-station and the national power grid [5]. The location is 160 km from Chittagong and 210 km from Dhaka, close to the Payra Port on the western bank of the Rabnabad River. This work was forwarded to Norinco International Power Ltd and the Rural Power Company Limited [6]. The main concern of this plant is its potential adverse effects on the local ecosystem and food supply; this power plant is regarded as one of the most contentious proposals [7]. Acid rain, smog, and harmful air pollution are examples of natural disasters brought on by coal-based power plants' significant contributions to carbon dioxide emissions and global warming [8]. Even though ultra-supercritical technology has since taken over this project, we could still determine the tolerance level and estimate the carbon emissions. However, Post-ignition carbon dioxide (CO_2_) capture (PCC) by amine absorption stripping is an ongoing technology for minimizing CO_2_ emissions [9,10]. One major obstacle to technological advancement is the solvent's suitability. When the process is in progress, the amine is degraded via irreversible mechanisms. Therefore, amine breakdown is an inevitable outcome. The degradation products threaten the environment, exacerbate corrosion in process equipment, create fouling, and eventually render the process inefficient, dangerous, and costly. Monoethanolamine (MEA) is the highly potential solvent utilized and evaluated at the industrial level for CO_2_ capture due to its power absorption rate, capacity to capture CO_2_, and medium to high resilience to oxidative and thermal degradation [11]. However, toxicity is a significant challenge in handling the MEA, which requires careful handling and safety protocols. So, scientists are looking forward to unique techniques for the mitigation of CO_2_. The thermodynamic modeling of the CO_2_ and potassium taurate solution system for the simulation of the CO_2_ capture process has developed. However, one significant challenge here is the variations in energy requirements in this process. Another one is an advanced fixed and capture level reduction operating mode for CO_2_ removal in a natural gas combined cycle power plant [12], which is suitable for the application of natural gas combined cycle (NGCC) where more investigation for coal power plants. A new deep eutectic solvent has been developed for CO_2_ capture: properties and applications in CO_2_ separation [[13], [14], [15]]. The mentioned new solvents have some drawbacks, namely the reaction environment's corrosive nature, the solvent's loss due to its volatility, and a high energy demand at the regeneration step [13]. However, the computerized carbon capture and storage system is fully technology-based and has no harmful interaction with the atmosphere.

Our research has enabled us to determine the prospective carbon emissions from the coal-based power plant using conventional production data subject to fluctuation. This allows us to analyze how our proposed computerized CCS technology would mitigate carbon leakage and protect the environment. A standardized methodology is utilized to compute emissions, which commences with the selection of an operational model that has been established and is specifically designed to estimate CO_2_ emissions from coal-based power plants. Despite efforts to implement ultra-supercritical technology, the project's carbon emissions remain a significant concern. This study aims to estimate the carbon emissions associated with the plant's operation and assess their potential impact on the surrounding area. Additionally, we propose the implementation of Computerised Carbon Capture and Storage (CCCS) technology, coupled with advanced computerized systems, to mitigate the environmental consequences of the plant's operations.

## 2 Methodology

A standardized process is used to calculate emissions, starting with the relatively typical step of choosing an operational model that has been established and is intended to estimate CO_2_ emissions from coal-based power plants. In order to maximize thermal output and reduce greenhouse gas emissions, start by selecting the best grade local coal (GHS). Although we used an operational model subject to change, we computed the findings using time (T) and load generation (G), as these are the parameters often used in coal-based power plants in Bangladesh. The carbon emission rate is represented by the product of O and Eco (carbon emission), obtained by multiplying both G and T [16]. Additionally, we employed basic chemical algebraic equations (gram molar mass basis) to compute the CO_2_ emission. Further carbon calculations will be more accessible with this dataset. The carbon sequestration of the plant area is calculated in the second step using global data on rates for different locations [17]. This technology must be briefly discussed, as it works to store carbon and greenhouse gases (GHGs) and permits their mitigation through reuse. To help with load generation and output power calculations, Table 2 offers an approximate dataset for calculating load percentage (L), load generation (G), and output power (O). The working day is divided into numerous sets of working hours. For the purpose of carbon sequestration, we consider the world's most extensive mangrove forest (Sundarbans) due to its proximity to the power plant. To calculate the capacity of the Sundarbans for carbon sequestration, we determined the region's area and the rate of carbon sequestration of the mangroves per area of land mass. Here, we have a short review and discussion of technology parallel to the power plant's carbon sequestration rate, and it also depicts the extent to which these specific technologies can be minimized. Finally, we developed the advanced computerized carbon capture and storage model, used the computer logical function to ensure carbon capture and storage.

### 2.1 Plant's operational model

**Fig. 1:**
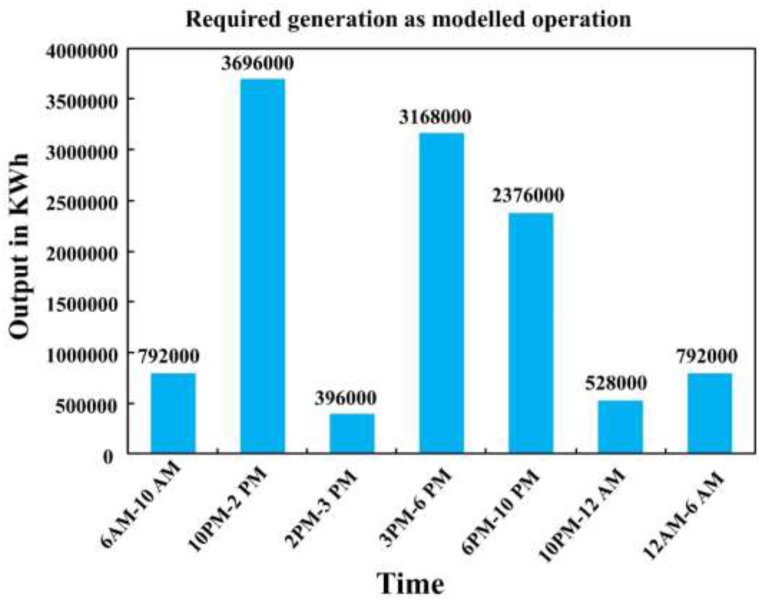
Time-segmented generation profile.

It is acknowledged that power plants often operate below their maximum capacity, with carbon emissions directly proportional to the load they generate [18]. There are typical daily generation profiles, which is the underlying assumption of this research. In this instance, the letters G, L, C, and T stand for generation, load, and capacity (1320 MW) of the power plant, respectively, and time (in hours). Multiplying the load by the capacity is the formula for calculating load generation. The output is expressed in kWh when the resultant load generation is multiplied by the time and F1000.The simplicity of this process allows for easy modification of settings and calculations with alternative data. Fig. 1 illustrates the required generation for different working hours, and Table 1.

**Table 1:** Plant operational model assumptions.

**Working (Hrs.)**	**T (Hrs.)**	**L (load)** %	Assumptions details
6 am–10 am	4	15	Minimum loaded time because of having closed some portion of power consumed sectors.
10 am–2 pm	4	70	Approximate fully loaded time for beginning period of the most sectors, which are the major part of the power consumed.
2 pm–3 pm	1	30	Medium loaded time because of resting some units, which also consumes power.
3 pm–6 pm	3	80	Peak loaded time due to fully active all power consumed sectors.
6 pm–10 pm	4	45	Medium loaded time because of resting some unit which, are the contributors of power consumed.
10 pm–12 am	2	20	Medium loaded time because of resting some units, which are the contributors of power consumed.
12 am–6 am	6	10	Minimum loaded time because of having closed some portion of power consumed sectors.
**Total**	**24**

**Table 2:** Modelled generation profile.

Working (Hrs.)	T (Hrs.)	L (load) %	**G (load generation) = L×C (Capacity) MW**	**O (Output) = G×T×1000 KWh**
6 am–10 am	4	15	198	792000
10 am–2 pm	4	70	924	3696000
2 pm–3 pm	1	30	396	396000
3 pm–6 pm	3	80	1056	3168000
6 pm–10 pm	4	45	594	2376000
10 pm–12 am	2	20	264	528000
12 am–6 am	6	10	132	792000
**Total**	**24**			**11748000**

### 2.2 Coal selection

The carbon dioxide emission factor is a critical component that dramatically affects the quality of coal used in coal-fired power plants, which affects emissions [19]. In addition, the generator's quality is quite essential. A critical measure of a generator's efficiency is its heat rate, which is required to create1 KWh of electricity; a lower heat rate denotes excellent performance. Multiplying the generator's heat rate by the CO_2_ emission factor and dividing the result by one million is how much carbon dioxide is created per kilowatt-hour [20]. Because of its comparatively low carbon dioxide emissions, sub-bituminous coal is considered a more environmentally benign choice.

### 2.3 Coal grade

**Fig. 2:**
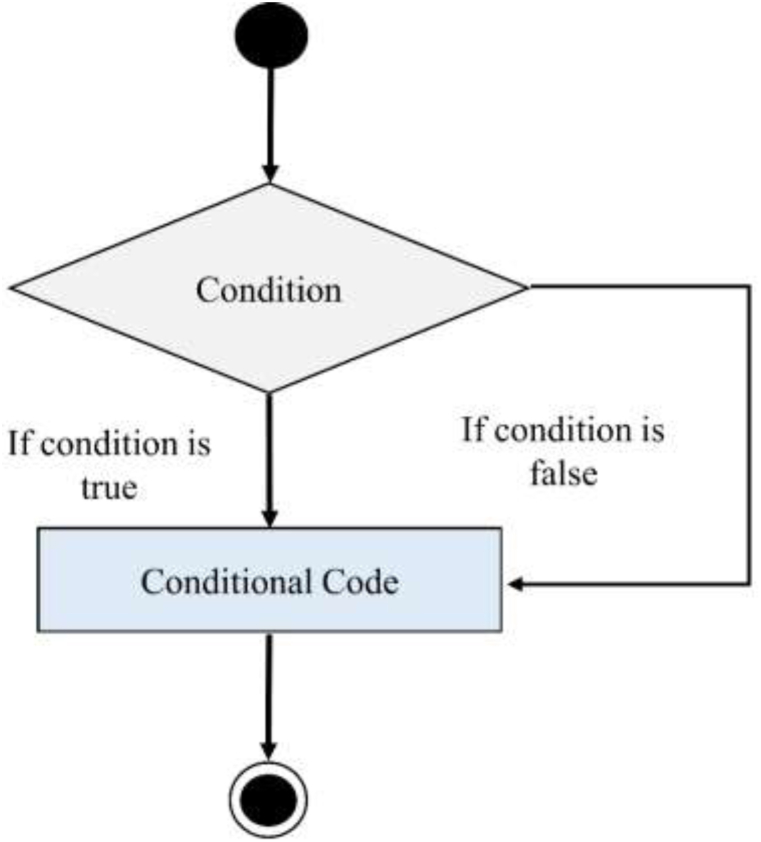
If-else structure [26].

The quality of coal significantly impacts the heat rate, with dry coal being preferred due to its ability to recover heat from flue gas. Coal drying equipment utilizes recovered heat to reduce coal moisture content, thus enhancing combustion efficiency [21]. Additional strategies for improvement include optimizing turbine cycles and boiler combustion efficiency [22]. Low-grade coal has a higher moisture content and produces more carbon dioxide when burned despite being more transportable and less expensive [23]. It is important to avoid using low-rank coal in power plants since using damp coal increases fuel consumption, flue gas flow rate, and auxiliary power consumption [24]. Therefore, thermal drying processes enhance plant productivity and boiler efficiency as well as reduce CO_2_ emissions. Furthermore, high-grade coal, known for its efficiency and low carbon content, is crucial for minimizing fuel processing costs in power plants [25]. The details assumption described as shown in below Fig. 2 [26].

• Capacity Factor: The capacity factor represents the electricity-generating efficiency and dependability of coal. In addition, higher capacity factors indicate that coal grades have higher efficiency.
• Heat Rate: A power plant's efficiency in turning coal energy into electrical power is determined by its heat rate; therefore, lower heat rates correspond to higher efficiency.
• Carbon Emissions: The amount of CO_2_ emitted per unit of energy generated by burning coal is known as carbon emissions. Coal grades with lower CO_2_ emission values are cleaner.

### 2.4 Advanced computer model for CCS technology

The proposed computerized model is explained programmatically with an if-else structure to analyze different conditions and circumstances in the results and discussion section. The “if-else" statement, contingent upon a given condition (test expression), serves as a pivotal decision-making tool in programming [[26], [27]]. It demonstrates the executed of specific code blocks based on whether the condition evaluates true or false. If the condition commands, the code within the 'if' block is performed; otherwise, the code within the 'else' block is executed [28]. This basic framework emulates situations in which decisions are made in the actual world and decisions guide future actions [29]. The decision-making statements are essential in programming because it may help determine the possibilities to be considered and how the program should be executed. Their ability to regulate program behavior and customize reactions to different situations gives programmers more control over the functionality and adaptability of software systems.

## 3 Result and discussion

### 3.1 Emission calculation

The carbon emissions can be determined by multiplying the output value (O) obtained from Table 2 which the carbon emission factor could be obtained from Table 3. Therefore, the resulting quantity of CO_2_ serves as a basis for calculating the implanted carbon content. Further, this process enables the estimation of carbon emissions associated with the specified output stimulating analysis and management of carbon footprints within the relevant context.

**Table 3:** Grade of coal [17].

Fuel	Capacity Factor C_f_ (%)	**Heat rate, H**_**R**_**(BTU/kWh)**	**Carbon Emissions (lbs CO**_**2**_**/MMBtu)**
Anthracite	85–95	10000	228
Bituminous	75–85	10500	205
Sub-bituminous	65–75	11000	212
Lignite	55–65	11500	215

### 3.2 CO_2_ emissions

**Fig. 3:**
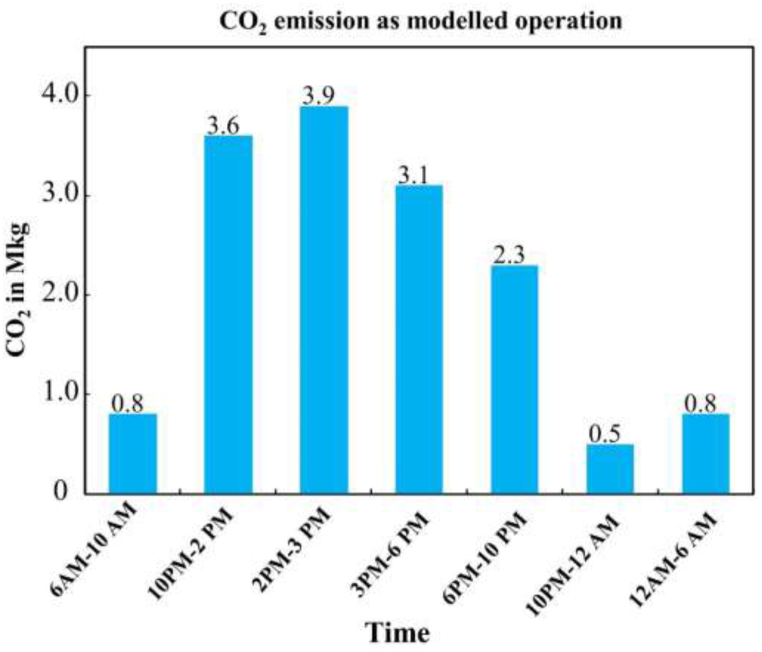
Plant emission by time interval.

The carbon emissions are determined by multiplying the output (O) obtained from Table 2 by the carbon emission factor (Eco) from Table 3, resulting in pounds of carbon emissions. To convert this value to kilograms (kg), it is multiplied by 0.453592 and then divided by 1,000,000, as one-pound equals 0.453592 kg (as shown in Table 4, column 5). Fig. 3 shows the carbon emissions from the plant based on the sported everyday generation profile during the period. The operation assumes a fixed load for a specified period, which enables analysis of the plant's carbon emission patterns.

**Table 4:** CO_2_ emission from modelled operation.

Time (Hrs.)	O (Output)	Eco (Carbon emission)	O × Eco lbs.	**{(O∗Eco)0.453592}/1000000 MKG.**
6 am–10 am	792000	2.16	1710720	0.8
10 am–2.0 pm	3696000	2.16	7983360	3.6
2.0 pm–3.0 pm	396000	2.16	855360	3.9
3.0 pm–6.0 pm	3168000	2.16	6842880	3.1
6.0 pm–10 pm	2376000	2.16	5132160	2.3
10 pm–12 am	528000	2.16	1140480	0.5
12 am–6 am	792000	2.16	1710720	0.8
**Total**	**11748000**		**25375680**	**15**

### 3.3 Calculation of carbon emission

It is necessary to know the amount of carbon in 11.6 K Mg of CO_2_ [30].

44 gm of CO_2_ = 1 mol.

1000 gm of CO_2_ = 1000/44 = 22.7 mol of C.

1 Mole of C = 12 gm of C.

22.7 mol of C = 22.7 × 12 = 272.4 gm of C.

1 kg of CO_2_ contains 272.4 gm of C = 0.2724 Kg of C.

15 kg of CO_2_ contains = 15V × 0.2724 = 4.086 kg of C.

The molar mass of CO_2_ = 44 gm/mole, gram molar mass of C = 12 [31].

So, 15 million kg of CO_2_ contains 4.086 million kg of carbon. As per our approximate generation profile, the Patuakhali power plant will produce nearly 4.086 million kg of carbon every 24 h of operation. Now, we need to find the amount of carbon Sundarbans can sequestrate. After our calculations, we ascertain that 15 million kg of CO_2_ contains approximately 4.086 million kg of carbon. Based on our estimated generation profile, it is evident that the Patuakhali power plant is projected to emit nearly 4.086 million kg of carbon during every 24 h of operation. Our next objective is to determine the capacity of the Sundarbans to sequester carbon.

### 3.4 Carbon sequestration

**Fig. 4:**
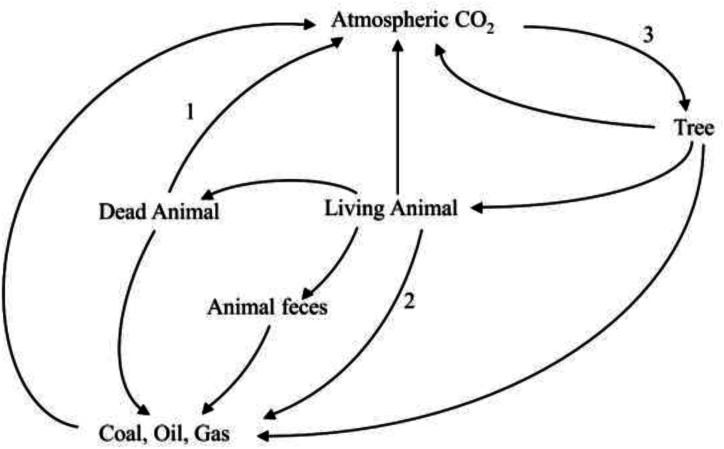
Carbon cycle and CO_2_sequestration process [35].

The natural carbon cycle encompasses various Earth components, including the biosphere, pedosphere, geosphere, hydrosphere, and atmosphere, facilitating the transfer and storage of carbon [32]. Carbon sequestration, which involves the absorption and retention of carbon in long-term reservoirs, plays a crucial role in mitigating the greenhouse effect and addressing climate change [33]. The goal is to sequester carbon dioxide from the atmosphere for extended periods, thereby reducing its greenhouse impact. Fig. 4 presents a detailed illustration of the carbon cycle during coal combustion, highlighting the interconnectedness of its components. The CO_2_ emissions enter the atmosphere and interact with water bodies during coal combustion. Through photosynthesis, plants and certain microorganisms absorb carbon dioxide from the atmosphere, converting it into organic compounds like sugar using sunlight for energy, thus facilitating their growth [34]. This process transfers carbon from the atmosphere to the biosphere. However, during nighttime, photosynthesis ceases, causing plants to release carbon dioxide as they respire. Additionally, all living organisms release carbon dioxide during the metabolic breakdown of organic compounds for energy [35].

Animals contribute to the carbon cycle by consuming green plants or other animals according to their dietary habits. Bacteria and fungi decompose their remains when organisms die, releasing carbon dioxide, water, and nutrients into the soil [36]. Water bodies also play a role in carbon assimilation, absorbing atmospheric CO_2_ and storing it as dissolved inorganic carbon. Phytoplankton in surface waters utilize carbon dioxide and sunlight for growth, with zooplankton [37] and microbes consuming phytoplankton and passing carbon up the food chain. This intricate process, encompassing terrestrial and aquatic ecosystems, is integral to the carbon cycle and carbon sequestration.

### 3.5 Approximate sequestration capability of sundarbans

Farmers provide intermediary firms with data on their practices to estimate additional carbon sequestration using advanced computer models, which undergo independent verification before being presented to clients [38]. However, the precise quantity of sequestered carbon remains to be determined due to the inherent imprecision of these calculations and Table 5 shown the approximate carbon sequestration of mentioned power plant area. This study focuses on the Sundarbans, a mangrove forest subject to tidal and storm influences, renowned for its ability to capture and retain fine sediment, organic matter, and coarse sediment. Compared to other forest types, the Sundarbans store significantly more carbon [39]. Mangrove sediments, rich in elements conducive to accelerated litter formation and increased sedimentation rates, also contribute to carbon storage [40]. Research indicates that mangroves can sequester approximately 25.5 million tons of carbon annually, equivalent to roughly 4.03 Pg (Pg) [41]. The Sundarbans area exhibits a carbon sequestration rate of approximately 3.7 pounds per acre per day [42]. The Sundarbans encompass 10,000 square kilometers, of which 70 % are in Bangladesh and the remaining 30 % are in India [43].

**Table 5:** Approximate carbon sequestration of Patuakhali per day.

Total area km^2^	Area in acre (A)	SQR (Sequestration rate)	Sq. (Sequestration)	A∗Sq	(A∗Sq)/1000000
**10000**	**2471,050**	**3.7**	**1.7**	**4200785**	**4.2**

### 3.6 Greenhouse gases (GHG) reduction technologies

The implementation costs associated with deploying these emission reduction technologies are substantial, necessitating equipment installation to filter exhaust/flue gases before their release into the chimney [44]. At [43], a comprehensive list of devices capable of controlling emissions from coal-fired power plants is presented in Table 6. Technological advancements have increased maintenance expenses for such equipment, often exceeding the initial purchase cost [45]. However, the primary objective of emission control systems is to mitigate the release of exhaust gases, particularly CO_2_ emissions, from power plants before they enter the environment. Unfortunately, many pollution control units lack modern analyzers capable of effectively capturing flue gases, including CO_2_, emitted by coal power plants, resulting in immediate release into the atmosphere.

**Table 6:** Summary of GHG control equipment and usefulness [45].

Targeted pollutants	Control equipment	Working principal	Percentage of removal
Particulate matter	ESP (Electrostatic precipitators)	The particles receive a charge through electrical induction. Next charged particles are gathered and drawn to various electrodes.	99.0–99.5 %
	Commonly refers to the fabric filter as a “baghouse.".	There is a cloth filter used. Flue gas is directed towards.	99.9 %
SO_2_	FGD (Flue Gas desulphurization unit (Commonly referred to as a 'scrubber')	A liquid sorbent, such as CaCO_3_, is injected or sprayed into the flue gas in wet FGDs. This takes up SO_2_ and turns it into a damp solid that can be disposed of and dried later.	80–99 %
NO_x_	Technologies for regulating combustion, like low-NO_x_ burners.	The coal combustion control unit is calibrated to reduce the amount of NO_x_ emissions.	40–45 %
	Controls used after combustion, such as selective catalytic reduction (SCR) and selective non-catalytic reduction (SNCR) units.	Ammonia is injected into the flue gas of SCRs. Next, water and nitrogen are created. The reaction is accelerated by the use of a catalyst.	70–95 %

**Fig. 5:**
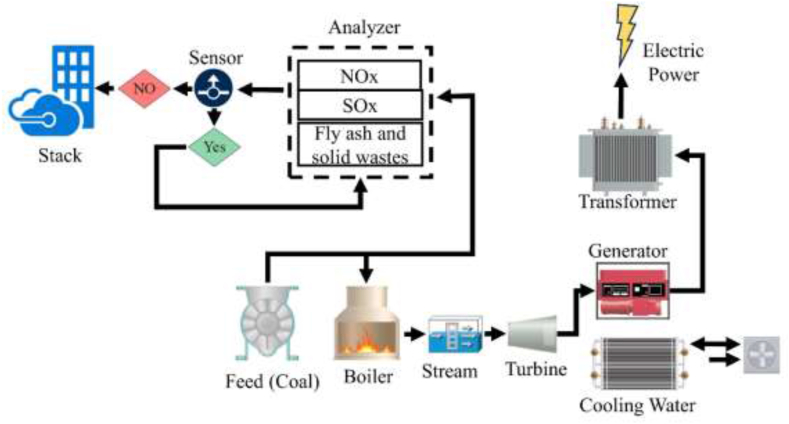
Computerized pollution control unit.

A computerized pollution control unit has been developed to address this, as depicted in Fig. 5. After combustion in the boiler, steam is directed towards a turbine for power generation, while the smoke containing flue gases is routed to a computerized analyzer. Before releasing the gases into the atmosphere, the analyzer scrutinizes them, triggering appropriate actions if exhaust gases are only partially captured. This iterative process ensures the effective capture of flue gases, mainly CO_2_, from coal-based power plants before their release into the environment.

### 3.7 Efficiency improvement for low-emission

**Fig. 6:**
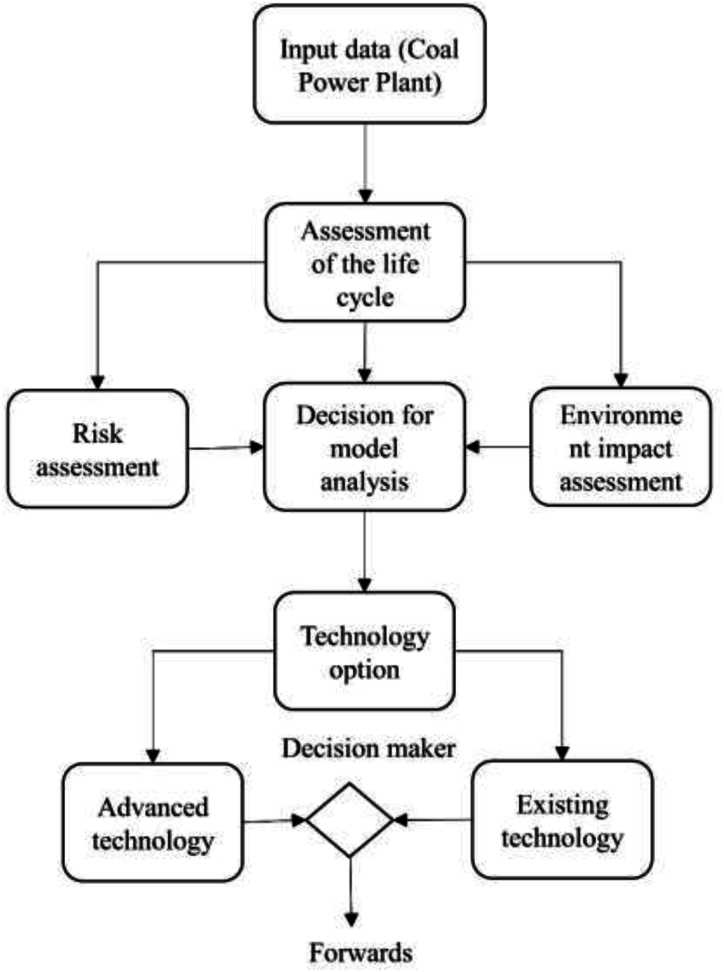
Efficiency improvement analysis for coal power plant.

**Fig. 7:**
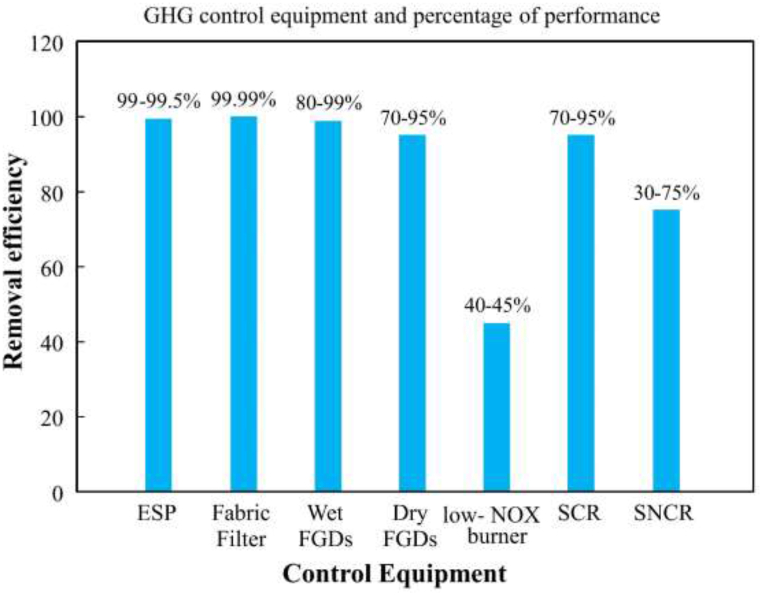
Performance of technology wise reductions.

It is essential to develop an efficient coal-fired power plant at the Patuakhali plant to reduce carbon emissions and variable costs. Energy audits and efficiency improvement assessments are two essential steps in this process. Inspection and rectification of faults must be done regularly to ensure plant efficiency. Studies suggest that pulverized coal-burning facilities can reduce their CO_2_ emissions by 2.7 % when their efficiency increases [46]. Additionally, increasing the heat rate is synonymous with enhancing efficiency and can significantly reduce emissions [47]. Coal-fired power plant automation plays an important role for lowering emissions and preserving the environment. The selection of appropriate technology is paramount for enhancing efficiency and reducing expenses. By implementing high-efficiency technology and automating the system, effective regulation of CO_2_ emissions can be achieved, leading to enhanced operational efficiency. Fig. 6 depicts the sequential stages of the coal-fired process, guiding the selection of state-of-the-art technology. Nonetheless, in order to reduce carbon emissions, three vital system assessments must be completed before manufacturing: the life cycle assessment, the study of air pollution emissions, and the environmental impact assessment [[48], [49]]. Additionally, risk assessment plays a pivotal role in decision-making. Thus, the selection of technological options depends on the co-existence of existing and advanced technologies, with favorable system analyses facilitating the adoption of cutting-edge technologies (see Fig. 7).

Table 7 illustrates the enhanced efficiency of technology-based alternatives for coal fuel. Meanwhile, Table 6 outlines potential strategies for improving heat rate and mitigating carbon emissions in coal-fired power plants. Lastly, Table 8 provides a comprehensive list of technological advantages derived from discussions on plant technology.

**Table 7:** Technology wise options for efficiency improvement [50].

Improvement option	Heat rate improvement (%)	Comment
Taking the moisture out of coal.	0.6 to 5.9	Considerably depends on coal type and heat is consumed.
Reusing heat.	1.2 to 3.6	Recuperating heat from exhaust or flue gas.
Enhancing the steam turbine loop.	2–4.5 s	Immutably founded.
Modernization of the heat rejection system.	0.5 to 3	Load specific.
Improving the auxiliary boilers.	0.2 to 1	In situ dependent.
Replacing and utilizing sophisticated controls and sensors.	Up to 1.5	Ageing dependent.
Coal flow regulation and balancing.	Up to 1	In situ dependent.
Optimization of combustion.	1 to 2	In situ dependent.
Enhancement of air blowing.	1 to 2	In situ dependent.

**Table 8:** Technology wise advantages [51].

Name	Advantages
Fluidized bed combustion	90 % reduction in the emissions of SO_x_ and NO_x_
Pulverized coal combustion systems	It is possible to achieve a 45 % efficiency level
Supercritical & ultra supercritical technology	Efficiencies could reach approximately 50 %
Integrated gasification combined cycle (IGCC)	Utilized is syngas, and leftover gases can be employed in other processes
Carbon capture and storage system	May absorb up to 90 % of carbon dioxide (CO_2_) without posing a health risk

### 3.8 Carbon capture, transportation, and storage system

**Fig. 8:**
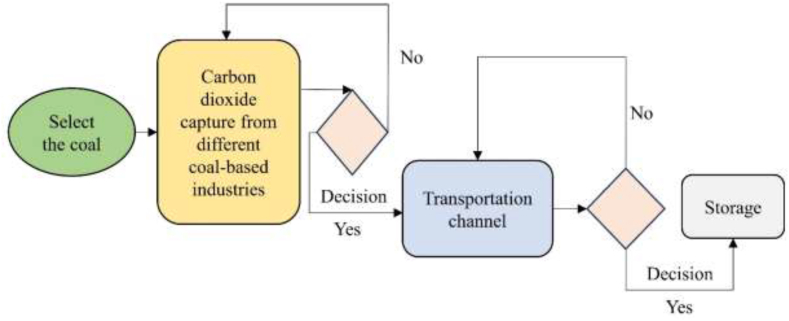
Computerized block diagram of CO_2_ capture, transportation, and storage system.

**Fig. 9:**
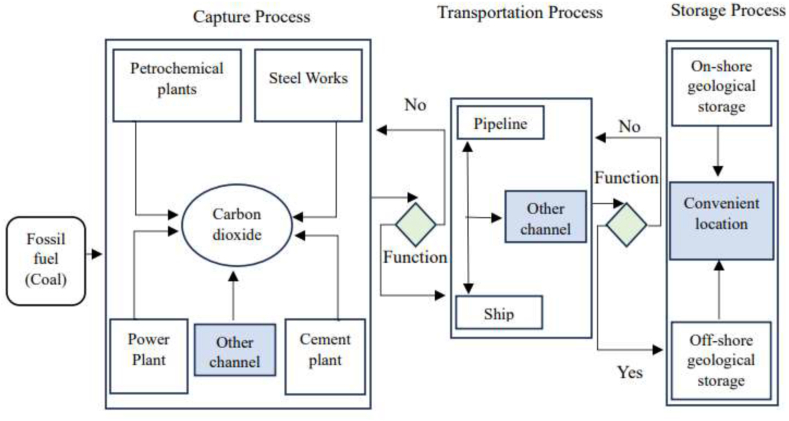
Basic block diagram of advanced computerized CCS technology.

Computerized carbon capture and storage (CCCS) is a highly efficient technology capable of capturing up to 90 % of carbon dioxide emissions from various industrial processes, thus preventing their release into the atmosphere [24]. Ensuring maximal capture of CO_2_ from coal-based power plants is paramount, yet technical malfunctions or unidentified issues may occasionally result in significant quantities of uncollected CO_2_. Although current CCCS technology achieves a capture rate of up to 90 %, efforts must continue to enhance capture capacity. Integration of an advanced CCCS system, as depicted in Fig. 8, is essential to optimize collection, transportation, and storage processes with built-in error detection mechanisms (Fig. 9).

Carbon capture can be accomplished through ox-fuel combustion, post-combustion, and pre-combustion capture methods [50]. This process typically involves three main steps: capturing CO_2_, transporting it, and securely sequestering it in depleted oil or gas fields and deep saline aquifers. After capture, millions of metric tons of CO_2_ are transported for commercial use or long-term storage. CCCS is highly efficient and capable of capturing up to 90 % of CO_2_ emissions without posing health risks, especially compared to super and ultra-supercritical technologies [51].

**Fig. 10:**
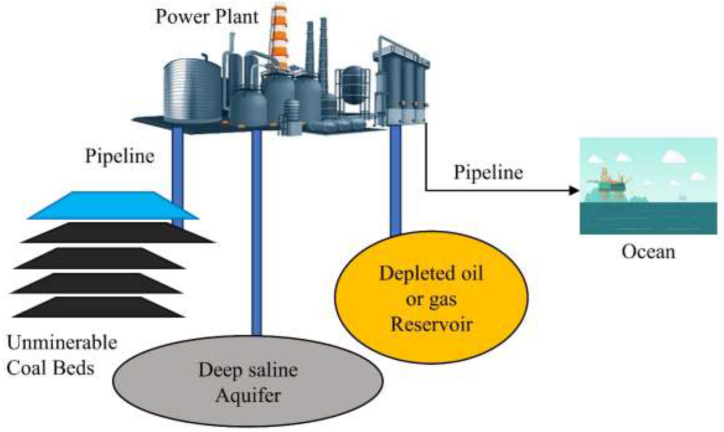
Burying carbon process.

CO_2_ captured from various industrial sources, including petrochemical plants, steel works, power plants, and cement plants, can be transported via pipelines, ships, or other channels for storage. Storage options include on-shore geological storage, off-shore geological storage, and other suitable locations as depict in Fig. 10. Captured CO_2_ can also enhance methane concentrations in coal seams, providing an additional benefit [52]. For fossil fuel power plants, capturing CO_2_ from flue gases presents a significant opportunity for emission reduction. Deep saline aquifers are particularly promising for long-term CO_2_ storage, while depleted oil and gas reservoirs offer potential for carbon capture, utilization, and storage initiatives [53].

## 4 Conclusions

The traditional methods for capturing CO_2_ using chemical solvents are relatively inexpensive and pose significant environmental risks. These approaches can lead to pollution and unintended chemical reactions in the atmosphere. While some solvents are specifically tailored for the natural gas combined power cycle (NGCPC), this technology is still evolving and has yet to reach its full potential. Conventional carbon dioxide collection equipment, such as electrostatic precipitators (ESPs) and flue gas desulphurization (FGD) systems, present significant drawbacks, including excessive water usage, high costs, challenges in waste management, and operational inefficiencies. To optimize these systems, various adjustments are required, such as reducing the moisture content in coal by up to 5.9 %, recycling heat by 3.6 %, enhancing the steam turbine loop by 2–4.5 %, and updating controls and sensors by up to 1.5 times. Our study estimates the carbon emissions from a coal-based power plant using conventional production data, which, despite some variability, provides a reliable baseline. By considering the sequestration rate of the Sundarbans, we have identified that natural processes can offset the carbon emissions from the Patuakhali power plant. Specifically, the plant's emission rate of 4.086 kg of carbon per day from 15 million kilograms of CO_2_ is minimal, yet it necessitates careful consideration and management. Computerized carbon capture and storage (CCS) technology is a promising solution. With the capability to reduce carbon emissions by more than 90 %, even accounting for a 10 % mechanical fault rate, CCS leverages advanced computational logic to prevent CO_2_ leakage and associated hazards. This approach is economically viable, requiring no additional costs or system improvements, and has zero negative environmental impact. The technique is especially appropriate for the geographical setting of the plant because it guarantees the security of the entire carbon capture, transportation, and storage process.

The Sundarbans are essential for maintaining biodiversity, and balancing environmental preservation and economic growth is critical. When fully operational, the integration of cutting-edge technology like automated CCS can reduce carbon emissions to almost zero. The Sundarbans can further enhance CO_2_ removal near the Bay of Bengal, underscoring the importance of combining advanced pollutant control technologies with CCS. Additionally, captured carbon can be utilized in enhanced oil recovery techniques within the petroleum industry, promoting environmental preservation and economic growth for future generations.

**Table:** Nomenclature

B	I
BTU = British thermal unit	IGCC = Integrated gasification combined cycle
C	K
C = Capacity	Kg = Kilogram
C_f._ = Capacity factor	kWh = Kilowatt-hour
CCS = Carbon capture and storage	L
CCCS = Computerized carbon capture and storage	L = Load
E	M
Eco = Carbon emission factor	MW = Megawatt
ESP = Electrostatic precipitators	O
F	O = Output
FGD = Flue Gas desulphurization	P
G	PSMP = Power system master plan
G = Generation	S
GHG = Greenhouse storage	Sq. = Sequestration
H	SQR = Sequestration rate
Hrs. = Working hours	
H_r._ = Heat rate	

## CRediT authorship contribution statement

**Md. Nasirul Islam:** Writing – review & editing, Writing – original draft, Project administration, Methodology, Formal analysis, Data curation, Conceptualization. **Md. Shameem Hossain:** Writing – review & editing, Supervision, Project administration, Formal analysis, Data curation. **Mohammad Mujtaba Hasan:** Formal analysis, Data curation. **Shehoba Yasmin:** Formal analysis, Data curation. **Prodeepta Neogi:** Formal analysis, Data curation.

## Data availability statement

All the data required are available within the manuscript.

## Declaration of competing interest

The authors declare that they have no known competing financial interests or personal relationship that could have appeared to influence the work reported in this paper.
